# Implementation of the virtual transitional care stroke intervention for older adults with stroke and multimorbidity: A qualitative descriptive study

**DOI:** 10.1177/26335565251323748

**Published:** 2025-02-26

**Authors:** Maureen Markle-Reid, Kathryn Fisher, Kimberly M. Walker, Jill I. Cameron, David Dayler, Rebecca Fleck, Amiram Gafni, Rebecca Ganann, Ken Hajas, Barbara Koetsier, Robert Mahony, Chris Pollard, Jim Prescott, Tammy Rooke, Carly Whitmore

**Affiliations:** 1 School of Nursing, 3710 McMaster University, Hamilton, ON, Canada; 2 Health Research Methods, Evidence and Impact, Faculty of Health Sciences, 3710 McMaster University, Hamilton, ON, Canada; 3 Aging, Community and Health Research Unit, 3710 McMaster University, Hamilton, ON, Canada; 4 McMaster Institute for Research on Aging, 3710 McMaster University, Hamilton, ON, Canada; 5 Upstream Lab, MAP Centre for Urban Health Solutions, 10071 St Michael’s Hospital, Unity Health, Toronto, ON, Canada; 6 Department of Occupational Science and Occupational Therapy, Rehabilitation Sciences Institute, Temerty Faculty of Medicine, 7938 University of Toronto, Toronto, ON, Canada; 7 Parkwood Institute, 25472 St. Joseph’s Health Care, London, ON, Canada; 8 Hotel Dieu Shaver Health and Rehabilitation Centre, St. Catherines, ON, Canada; 9 169551 CarePartners, Kitchener, ON, Canada

**Keywords:** Older adults, multimorbidity, stroke rehabilitation, transitional care intervention, implementation

## Abstract

**Background:**

Older adults with stroke and multimorbidity experience frequent care transitions, which are often poorly coordinated and fragmented. We conducted a pragmatic randomized controlled trial (RCT) to test the implementation and effectiveness of the Transitional Care Stroke Intervention (TCSI), a 6-month, multi-component, evidence-informed intervention to support older adults with stroke and multimorbidity using outpatient stroke rehabilitation services. The TCSI was designed to support self-management, improve health outcomes, and enhance the quality and experience of care transitions.

**Objective:**

To explore the facilitators and challenges to implementing the TCSI, from the perspective of healthcare providers (HCPs) (n = 12) and Managers (n = 3).

**Methods:**

Data collection and analysis were guided by the Consolidated Framework for Implementation Research (CFIR). Data were collected from study documents, individual and group interviews conducted with HCPs and a Care Coordinator, and surveys from managers. Data were analyzed using thematic analysis.

**Results:**

Intervention implementation was facilitated by: a) strong collaborative and interdependent HCP team relationships, b) dedicated resources (funding, staffing) to support intervention delivery, c) training and ongoing support, customized to individual HCP needs, d) organizational readiness, strong leadership, and effective champions, e) structures to facilitate virtual information-sharing, and f) regular monitoring of intervention implementation. Implementation challenges included: a) COVID-19 related challenges (staff turnover, community service disruptions), b) poor communication with community service providers, c) documentation burden (intervention-related), and d) virtual care delivery.

**Conclusions:**

This research enhances understanding of the diversity of factors influencing implementation of the TCSI, and the conditions under which implementation is more likely to succeed.

## Introduction

The burden of stroke in Canada is expected to rise due to an increase in the number of people experiencing and/or surviving a stroke, which is a consequence of various forces including population growth, aging, and improvements in acute stroke care. Alongside improvements in acute stroke care services has been relatively short hospital length of stays in most developed countries; thus, most of the stroke patients’ recovery occurs after the patient is discharged from the hospital.^1^ Up to 60% of older adults hospitalized with a stroke are discharged directly home after an acute care or in-patient rehabilitation hospital stay, and up to 60% will require ongoing rehabilitation in the community that supports continued recovery and reintegration into the community.^2^

The substantial emotional, social, and health-related challenges^3^ experienced after hospital discharge are complicated by health care transitions, with older people with stroke experiencing a median of 3 and as many as 7 transitions after discharge from acute care in the first 90 days post-stroke.^4^ Transitions refer to the movement of patients across different healthcare settings, locations, providers, or levels of care, and are “marked by changes in patients’ physical, mental, emotional, and cognitive capacities”^5,6^(p. 790). The goal of transition management is to facilitate and support seamless movement across the continuum of care and achieve and maintain optimal outcomes and quality of life for patients.^5,6^ Poor quality transitions are common and associated with avoidable hospital readmissions, increased healthcare costs, reduced quality of life, reduced patient satisfaction and safety and increased burden on family caregivers.^7,8^

Stroke survivors are often unable to access essential rehabilitation services following hospital discharge.^9–11^ In Ontario (Canada), many stroke patients do not receive the required intensity of rehabilitation therapy recommended by the Canadian Stroke Best Practices (received 69 minutes/day compared to the recommended 180 minutes/day), and only 50% of stroke patients access post-acute (inpatient and/or community-based) rehabilitation.^12^ A meta-ethnographic systematic review attributed limited access to community services and primary care support to the feelings of abandonment that stroke survivors and their caregivers report following hospital discharge.^10,13^

Older adults with stroke arguably bear the brunt of these deficiencies, given that up to 80% of all strokes occur in older adults. Moreover, the vast majority (92%) of older adults with stroke have multimorbidity (≥ 2 co-morbidities).^14,15^ Multimorbidity has been linked to lack of care coordination/communication in transitions across care providers.^5^ Accordingly, older adult stroke survivors tend to be a more medically complex subgroup that face increased short- and long-term mortality, poorer health status, lower baseline functional status, longer hospital stays, and higher use of health services compared to younger stroke survivors.^15,16^ These problems with transitional care will continue to escalate with the number of older adults with stroke and multimorbidity expected to increase by one-third by 2035.^17^ Consequently, TC services and longer-term support are priority issues for older adults with stroke and multimorbidity.

There is broad consensus that better-coordinated transitions, which in part can be accomplished by improved access to post-acute stroke rehabilitation services and community resources, are urgently needed^18,19^ to address the deficiencies in transition quality.^20–23^ However, there is little evidence about the extent to which TC interventions can prevent adverse outcomes, which components of TC interventions are most effective, and for which outcomes, and which populations are most likely to benefit.^24,25^ Current Canadian best practice guidelines for managing care transitions following stroke are largely built upon evidence from observational or qualitative studies, or expert consensus.^5,20^ Moreover, while transitional care interventions have been recommended to improve hospital-to-home transitions in community-living older adults with complex needs^26–30^ and have the potential to improve care for those with stroke,^26–29,31,32^ none have been developed specifically for older adults with stroke and multimorbidity, leaving uncertainty about how best to achieve optimal outcomes in this population. Stroke in the context of multimorbidity has important implications for the design of stroke health services.^33^ Older adults with stroke and multimorbidity are more likely to experience treatment burden,^34^ poorly coordinated care and service fragmentation because of the complexity of their healthcare. There is a need for integrated TC interventions that provide the holistic, person-centred care needed to improve the experience and outcomes of this complex and increasing subgroup of the stroke population.^15^

Another limitation of the existing evidence for TC is that the vast majority have focused on hospital-based initiatives such as early supported discharge interventions,^11^ with very few examining the role of outpatient or community-based stroke rehabilitation teams in supporting care transitions, despite 65% of community-based stroke rehabilitation being provided through outpatient stroke programs.^12^ Furthermore, the implementation and experience of virtually delivered transitional care – a mode of delivery that became increasingly relevant due to the COVID-19 pandemic – has focused on evaluating effectiveness^35^ and user satisfaction^36–38^ rather than implementation. The Canadian Stroke best practice guidelines for managing care transitions following stroke recommended more research on how to best provide TC interventions to enhance transitions across care environments for older adults with stroke.^5^

The Transitional Care Stroke Intervention (TCSI) is a new, patient-centred delivery model designed to support self-management and improve the quality and experience of transitions. The TCSI was implemented in a multi-site pragmatic randomized controlled trial (RCT) to address the gaps in post-acute TC for older adults (≥ 55 years) with stroke and multimorbidity (ClinicalTrials.gov: NCT04278794). We previously reported the main trial findings on the effectiveness of the TCSI,^39^ which showed no effect from the TCSI on the primary outcome of hospital readmissions, but significant improvements in the secondary outcomes of physical functioning, stroke self-management and patient experience. There was no effect from the TCSI on the secondary outcome of health and social service use costs.^39^

It is important that the TCSI is evaluated to assess its effectiveness. However, evaluating implementation of the TCSI in real-world practice is equally important.^40,41^ A comprehensive implementation evaluation can help to interpret trial results and determine how delivery of the intervention can be optimized to effectively spread it to other jurisdictions.^41^ The main research question for the implementation component of the evaluation was: From the perspective of healthcare providers (HCPs) and managers, what are the facilitators and challenges to implementing the TCSI for older adults with stroke and multimorbidity using outpatient stroke rehabilitation services?

## Methods

This multi-method qualitative study was embedded within a pragmatic RCT that evaluated the implementation and effectiveness of the TCSI. The study design was a Type II hybrid implementation-effectiveness study^39^ where implementation and effectiveness are given equal weight.^42,43^ The details regarding the intervention and study methods, including modifications to the original protocol triggered by the COVID pandemic, are provided in our published paper,^39^ thus are only briefly summarized here. Following this, the methods associated with the implementation study are provided.

### The transitional care stroke intervention

Building on a previous feasibility study,^44^ the TCSI was tested in two local hospital-based outpatient stroke rehabilitation programs within two geographical areas in Ontario, Canada (Hamilton and Niagara). In addition to receiving usual care offered through their outpatient stroke rehabilitation program, the TCSI involved virtually delivered transitional support by an interprofessional (IP) team of HCPs from a hospital-based outpatient stroke rehabilitation program over a 6-month period following randomization. The IP team members (referred to as “participants” below) included an Occupational Therapist (OT), Physical Therapist (PT), Speech Language Pathologist (SLP), Social Worker (SW), and a Registered Nurse (RN) or Registered Practical Nurse (RPN). The Physical Therapist or Occupational Therapist also functioned as the Care Coordinator/system navigator on the teams. To avoid contamination with the control arm, all providers that were members of the intervention team did not deliver usual care to participants in the control arm of the study.

The core components of the intervention were based on evidence-based guidelines to prevent and manage stroke,^5,20,45,46^ multimorbidity,^47,48^ and transitions in care,^20^ and were designed to support self-management. . The intervention comprised both fixed and flexible components that were tailored to the local context in collaboration with the HCPs and managers who delivered the intervention. This approach is in keeping with research that suggests that interventions should be standardized by the function of a component, rather than standardizing interventions according to the specific form that they take.^49^

The foundational component of the TCSI was post-acute care coordination and system navigation support provided by a designated member of the IP team who also served as a Care Coordinator. The Care Coordinator contacted the older adult study participant by phone two days post-discharge and conducted the first virtual visit by phone or videoconference within 14 days of discharge. The other components of the intervention included: 1) up to 6 virtual visits delivered by phone or videoconference by a member of the interprofessional (IP) team that were an average of one hour in duration (first visit occurred within 14 days of discharge and then monthly thereafter), 2) monthly virtual IP team conferences of the intervention team where the team developed an individualized patient-centred plan of care, co-developed along with the older adult, and engaged in ongoing evaluation of the plan of care at each team conference, and 3) an online resource to support self-management and system navigation (My Stroke Recovery Journey website (MSRJ).^50^ The IP team’s main activities during the virtual visits included: 1) conducting a comprehensive assessment of the health and social care needs of older adult participants using standardized valid assessment tools^5^; 2) use of evidence-based guidelines to prevent and manage stroke,^5,20,45,46^ multimorbidity,^47,48^ and transitions in care^9^; 3) conducting medication review and reconciliation and supporting medication management using best practices^51^; 4) providing self-management education and support using a strengths-based approach^52–55^; 5) providing care coordination and system navigation support; 6) providing education and support for older adults’ ongoing use of the MSRJ Website to support system navigation; 7) assessing and supporting caregivers; and 8) communicating alerts to primary care providers regarding the presence of dementia, depressive symptoms or medication issues in participants. The providers had access to two shared information systems (SharePoint and Electronic Medical Record) for virtual communication and information sharing within the team and documenting the care that they provided.

The intervention included a number of elements from the National Institute for Health and Care Excellence (NICE) guideline, which focuses entirely on the assessment and management of multimorbidity, including recommendations on: 1) person-centred care (e.g., considers individual’s needs, preferences for treatment, health priorities, lifestyle and goals), 2) comprehensive assessment that includes assessment of the social determinants of health using validated assessment tools, 3) assessment of treatment burden (how their health problems, individually and collectively, affect their day-to-day life), 4) IP team of HCPs working together to provide integrated care, 5) individualized plan of care that takes into account the individual’s multiple chronic conditions, medications, and the social determinants of health, 6) medication review (identification of potential interactions), 7) care coordination to ensure continuity of care across different providers and settings and prevent gaps in care, and 8) emphasis on health education and self-management (shared decision-making between the individual and the health care team, with the individual playing an active role in determining their treatment plan and goals).^48^

### Overview of the pragmatic trial

Study participants were recruited from two local hospital-based outpatient stroke rehabilitation programs by trained recruiters. Study recruitment was conducted during 2020-2021 and spanned 11 months. To be eligible, individuals had to: 1) be aged 55 years or older, 2) have a confirmed diagnosis of stroke (first ever or recurrent), 3) be referred to an outpatient stroke rehabilitation program, 4) have at least two self-reported chronic conditions in addition to stroke, 5) be planned for discharge from hospital to the community (not long-term care), 6) have access to technology to participate in virtual visits, (e.g., access to a telephone, tablet, or other device with video capabilities, and internet connection), 7) be competent to provide informed consent, as determined by a score of 18 /22 on the T-MoCA^56^ or have a substitute decision-maker who could provide consent on their behalf, 8) speak or understand English or have an available interpreter, and 9) reside in one of the two participating regions with no plans to move out of the region during the trial. Multimorbidity was defined as two or more chronic conditions that included the following categories: cardiovascular, respiratory, mood disorders, gastrointestinal, endocrine, liver, kidney and urogenital disorders, hearing and vision, neurological, musculoskeletal, pain, substance abuse, infection. These conditions were chosen because they are prevalent in older adults, and are frequently reported in the literature on multiple chronic conditions^57^ Participants were asked to self-report their diagnosed chronic conditions. Each chronic condition counted in terms of multimorbidity, so, for example, if a participant had two different cardiovascular conditions (e.g., hypertension, atrial fibrillation), each one counted so was considered as two conditions for the purpose of identifying multimorbidity. A full list of the chronic conditions that participants self-reported are outlined in Supplement Appendix 1.

Study sites were diverse with respect to geography (one site was urban/rural, one site urban), socioeconomic, and ethnocultural characteristics. The Hotel Dieu Shaver Health and Rehabilitation Centre is a separate rehabilitation centre (Site A) within the Niagara region in Ontario. Hamilton Health Sciences Regional Rehabilitation Centre is in a large tertiary teaching hospital, directly connected to an acute medical hospital, the Hamilton General Hospital (Site B). Each site had an IP team of healthcare providers, including a 0.80 full-time equivalent Care Coordinator, who delivered the intervention. Part of the IP team at Site A (Occupational Therapist, Physical Therapist and Speech Language Pathologist) had a pre-existing working relationship as members of the outpatient stroke rehabilitation team. The IP team at Site A provided both the TCSI and usual outpatient stroke rehabilitation services to participants randomized to the TCSI. The intervention team in Site B was composed of HCPs drawn from different hospital units (not outpatient stroke rehabilitation) that was formed specifically for this study. The intervention team providers in Site B liaised with a separate outpatient team providing usual outpatient stroke rehabilitation services.

The study paid the participating outpatient stroke rehabilitation sites to deliver the intervention, to compensate the staff that were delivering the intervention, and offset other material costs associated with the delivery of the intervention. The trial was initiated 7-8 months after the COVID-19 pandemic started in Canada (March 2020). Of the 90 older adults enrolled in the trial, 44 were randomized to the TCSI (intervention group), and 46 were randomized to usual care (control group). Of the forty-four intervention participants enrolled at baseline, 93% (41/44) received at least one visit by a member of the IP team.

### Implementation evaluation

We used a qualitative descriptive approach, as described by Sandelowski,^58,59^ to identify the facilitators and challenges to implementing the TCSI. A qualitative descriptive approach provides a fulsome summary while remaining close to the words of participants when describing their experiences implementing the TCSI.^58^ Data collection and analyses were guided by the Consolidated Framework for Implementation Research (CFIR) framework.^60^ CFIR is an implementation determinant framework^61^ designed to describe challenges and barriers to implementation outcomes (adoption/adoptability, implementation/implementability, sustainment/sustainability), which in turn shape the ultimate success of the intervention (achievement of intervention outcomes).^60^ This framework consists of 39 constructs, organized into five domains known to affect implementation of interventions: 1) individual characteristics (including their knowledge and beliefs about the intervention, stage of change, and personal attributes); 2) innovation characteristics (adaptability, complexity, and design quality); 3) outer setting, which includes external factors (e.g., political, social, economic); 4) inner setting (e.g., organizational factors such as structures, communication, and readiness for implementation); and 5) implementation process including planning, engagement of key players, and how the intervention is executed.^60^

CFIR was selected over other implementation determinant frameworks for the following reasons: 1) CFIR is one of the most widely used implementation frameworks in the field of implementation science,^62^ 2) CFIR is a comprehensive framework that includes a wide range of determinants that can influence implementation success. It considers factors at the individual, organizational, and external levels, providing a holistic view of implementation processes that are grounded in empirical evidence,^62^ and 3) CFIR provides a practical guide for implementation planning and evaluation. It includes constructs that can be measured and assessed, helping to identify areas for improvement and track progress over time. The Consolidated Criteria for Reporting Qualitative Research (COREQ) Checklist guided reporting of the methods, context, findings, analysis, and interpretations of the study^63^ (Supplement Appendix 2).

### Participants

This study focuses on the implementation of the TCSI with HCPs and Managers who delivered the intervention. Study participants were HCPs involved in two IP teams (a total of 12 individuals), and a total of three managers from the two study sites. All IP team members were invited to participate in the study. There were no exclusion criteria.

### Data collection

#### Demographic characteristics of health care provider and manager participants

Demographic characteristics of the HCPs and Managers involved in delivering the TCSI intervention were collected prior to focus groups and interviews, including age, education, discipline type, years working in their discipline, and years working in their current position.

#### Document review

We analysed study documents associated with TCSI implementation including meeting minutes from: 1) Care Coordinator meetings attended by the PI, RC, and Care Coordinators within each site, and 2) outreach meetings, attended by the PI, RC, and IP team members and their managers within each site. A total of 32 documents were reviewed. Meeting minutes were recorded by the RC.

#### Focus groups and interviews

All members of the IP team within each of the sites were invited by the RC by e-mail to participate in two focus groups conducted by a female research assistant (RA) at 6- and 14-months (4 in total, online using zoom) following initiation of the intervention (1½ hours). The RA (BScN) was trained in qualitative methods, blinded to the goals of the intervention, and not previously known to the participants. A Care Coordinator from one of the sites also participated in an interview at the end of the study recruitment period. The research team developed focus group and interview questions, guided by the CFIR framework,^60^ to identify the facilitators and challenges of implementing the TCSI. The focus group and interview questions were piloted by the PI and the RC with one HCP recruited from the IP team. All focus group and interview participants received the interview questions in advance to review.

#### Surveys

All managers responsible for the IP teams from the two study sites were invited by the RC by e-mail to complete a semi-structured survey at 6- and 14-months (2 in total, online using Survey Monkey) following initiation of the intervention. A total of 6 surveys from 3 Managers were received. The survey questions were open-ended versions of the focus group questions.

### Data analysis

Descriptive statistics were used to summarize the characteristics of the HCP and Manager participants. All audio recordings of qualitative data (interviews, focus groups) were transcribed verbatim, and then the transcript was checked for accuracy against the recording and anonymized. All documents there then uploaded and organized using NVivo Version (QSR International) software version 12.Themes were generated using thematic analysis, which is consistent with a qualitative descriptive approach.^58^ Thematic analysis involved using deductive coding with the CFIR framework and inductive analysis for sub-codes within the CFIR domains. This analytical approach allowed us to develop an understanding of the factors that served as facilitators and challenges to implementation of the TCSI.

An external consultant (RV), not involved with data collection, with experience in qualitative research methods and CFIR application, independently analysed all interview and focus group transcripts. Analysis was completed line-by-line applying a priori codes of the CFIR domains and constructs. In addition to the consultant, the PI independently coded two focus group transcripts to ensure inter-rater reliability in coding. After this, a coding framework was developed and finalized in discussions with the research team and applied to the remaining transcripts. As analysis proceeded, the PI, RC and external consultant met regularly to review the coding and identify relationships between codes, further combine or collapse codes, discuss emerging ideas, and identify discrepancies, which were resolved through consensus. Data from the study documents and surveys were reviewed and analyzed by the consultant, PI, and RC. These data were triangulated with focus group and interview findings. Themes were then shared with the research team, to ensure they were reflective of the data.

### Rigour and trustworthiness

Multiple strategies were used to enhance the rigour and trustworthiness of the findings. An audit trail was developed to document all decisions and procedures related to recruitment, data collection and analysis. Consensus was reached by all authors prior to the inclusion of themes in the final report. Transcripts from the focus groups and interview were coded by multiple team members. Investigator triangulation was used in data analysis through team meetings with several research team members with expertise in qualitative research, use of CFIR, older adults, and community-based interventions. Conflicts were resolved through team consensus. The study sample and setting were described to enhance transferability.^64^

### Ethics approval and consent to participate

The study was conducted in accordance with the Tri-Council Policy Statement, Ethical Conduct for Research Involving Humans.^65^ Institutional ethics approval was obtained from: the McMaster University Hamilton Integrated Research Ethics Board (REB) (# 8017) and the Hotel Dieu Shaver Health and Rehabilitation Centre REB, and renewed yearly, as required. Operational approval to conduct the study was obtained from each program site. All focus group and interview participants provided written informed consent prior to data collection.

## Findings

### Demographic characteristics

The demographics of the 12 HCP participants are displayed in Table 1. The three manager participants had between 3 and 21 years of experience in their current position; two had a physical therapy background; one had an occupational therapy background; two had a bachelor’s degree, and one had a master’s degree.

**Table 1.** Demographic characteristics of healthcare provider participants (n = 12).

Characteristics	Category	N	%
Sex	Female	11	91.7%
	Male	1	8.3%
Age (years)	26-40	6	50.0%
	41-60	6	50.0%
Education	Diploma, Bachelor’s degree	4	33.3%
	Graduate degree	8	66.7%
Discipline	Registered Nurse	3	25.0%
	Occupational Therapist	3	25.0%
	Physical Therapist	2	16.7%
	Social Worker	2	16.7%
	Speech and language Pathologist	2	16.7%
Years working in discipline	0-10	7	58.3%
	11-20	2	16.7%
	21+	3	25.0%
Years working in current position	0-10	8	66.7%
	≥ 11	4	33.3%

### Themes

CFIR-driven analysis of the data generated 10 themes, which included six factors facilitating implementation and four factors challenging implementation. Facilitators promoted the implementation of the core components of the intervention while challenges hindered the implementation of the core components of the intervention. These themes spanned the five CFIR domains (Innovation Characteristics, Outer Setting, Inner Setting, Characteristics of Individuals, and Implementation Process). The most reported domains impacting implementation were in the inner setting (7 facilitators and challenges), intervention characteristics (5 facilitators and challenges), and implementation process (4 facilitators and challenges), while factors in the outer setting and individual characteristics were less reported. Table 2 provides a summary of the themes organized by implementation facilitators and challenges across the CFIR domains as well as exemplar quotes.

**Table 2.** Qualitative themes, organized by implementation facilitators/challenges across the CFIR domains.

Implementation Facilitators	CFIR Domain	CFIR Construct	Exemplar Quotes
Strong collaborative and interdependent HCP team relationships	• Innovation characteristics • Inner setting • Implementation process • Individual characteristics	• Innovation design • Relational connections • Teaming • Implementation team members	• I think that was definitely a benefit of this study, is that we’re all familiar with each other. we’ve all developed those relationships, we’re all very knowledgeable about each other’s roles. [HHS01] • We worked well as a team, I think, and just increased communication and interdisciplinary conversations and getting more comfortable with that. [HDS01]
Dedicated resources (funding, staffing) to support delivery of the intervention.	• Inner setting • Outer setting	• Available resources • Financing	• To implement the study - yes we had/have sufficient resources. However, moving forward after the study, we do lack the funding needed to carry on with this type of programming (managers survey) • There would need to be significant investment to support the increased resources required for this model of care (managers survey)
Extensive training customized to individual HCP needs and ongoing support provided by the research team.	• Innovation characteristics • Inner setting • Implementation process	• Innovation design • Access to knowledge and information • Assessing needs (innovation deliverers) and planning	• … how prepared I felt delivering the program because coming in later I got the training and I think it was well done. I think it was extensive to make sure we felt we were understanding of the program, what they’re trying to achieve, and then look practically at what we’re doing in outcome measures and stuff like that. I thought that was well done, because when I did my training, I was ready and rolling quick. I think that is a reflection of the training and support that we got from the research team and from my peers in helping support me to get up and running quickly. So, I think that was well done. [HHS06] • I felt like the Research coordinator was always available if there was an issue for me to either emailed her or call her and she was quick to reply, and I was grateful for that throughout the entire study. [HDS04]
Organizational readiness, strong leadership, and effective local champions	• Inner setting • Individual characteristics	• Mission alignment • Implementation leaders	• The program fits very well-with the additional staffing funded for the study (managers survey)
SharePoint and monthly IP team conferences facilitated virtual information-sharing and IP collaboration	• Inner setting	• Information technology and communications	• I felt like you [addressing Care Coordinator] had this big picture. I felt I was doing my piece, and then in case conference you were able to bring it together and often you were quite familiar with most of the patients. Whereas as a [profession] I’m only seeing a handful, and so you were often able to give good suggestions around resources or who/ what discipline made sense to maybe follow up next. (B2) • And that collaboration site that HDS04 organized all that, the folders and everything …. I can’t imagine having been able to do our job without that. [HDS01]
Regular monitoring of intervention implementation, feedback mechanisms and flexibility embedded in implementation.	• Implementation process • Innovation characteristics	• Reflecting and evaluating (implementation), adapting • Innovation adaptability	• The feedback on the intervention is important to the implementation evaluation. It can also help to identify and resolve any issues with delivering the intervention. [Care Coordinator Meeting]
Implementation challenges			
COVID-19 related challenges: Recruiting and retaining intervention staff and disruptions to community-based services and supports	• Implementation process • Inner setting • Outer setting	• Engaging intervention deliverers • Available resources • Critical incidents	• Many times, I felt like I was wearing many hats, a little bit of social work, a little bit of this a little bit of that, especially when our initial coordinator HHS01 HHS01, was gone and before our OT and coordinator HHS06 was back into the study, I felt like I was kind of like PT and OT. (B2) • Previous to that, right [HDS01], we had a program in our community that, through the region, that stopped. And it was very well populated by people leaving our direct outpatient care, for exercise. And before that, we had a link with heart Niagara, which was the local cardiac rehab provider. This is years and years ago, but then they changed ownership, when the LHIN came in, they changed ownership of cardiac rehab to another program that was inappropriate for us to refer to. So, it’s always moving, and even if somebody announced something that was great coming along, I’d a little bit think, “well okay great, but how long is this one going to last too? [HDS05]
Poor communication and collaboration with local community-based service partners	• Outer setting	• Partnerships and connections	• Yeah. One thing though, [HDS01] had sent out this post about all these different community services. I had no idea that there were so many community services, so I haven’t been able to use it, that information, yet in this study. But it makes me more aware for other patients on my units, or even in my family or personal life like, “Hey, you’re talking about a situation I might be able to find a community assistance program that might benefit you,” … [HDS03]: • I think what would be crucial to deliver this intervention is buy-in and partnerships with home and community care. I don’t think they were part of this study or part of this conversation, and they would be very crucial component to be able to successfully continue to deliver this intervention (A2)
Documentation burden (intervention-related)	• Inner setting • Innovation characteristics	• Available resources, compatibility • Design quality and packaging	• … it’s good to do outcome measures […] but I feel as though that took away greatly from the time that I was actually able to facilitate my interventions. I felt very scattered a lot of the time. I’m jumping between a stroke safety checklist, then to falls risk, then to community reintegration. [They are] very cumbersome, very wordy, super time-consuming, when at the core of this, these patients aren’t as interested in their outcome measures, as we are. It’s more about the care for them. (B5) • … the first 45 minutes is going through Falls, the PHQ2, TCSI, the Visit Record […] there’s like seven of them, or eight - the stroke safety checklist. […] so then, when I get to the actual discussion of the goals, […] behavioral change, all these things, it gets to the point where they are so tired of asking and answering these ridiculous questions that they just want to get to work on their intervention. (B3)
Technological/Virtual challenges impeded virtual delivery of the intervention.	• Innovation characteristics	• Design quality and packaging	• Technology challenges - at times, many technology challenges (sometimes provider issue, sometimes patient issue, sometimes internet issue) leads to much wasted time for both staff and patient (managers survey) • … as a physiotherapist, you know, some people might have balance problems. It’s very hard to assess and progress balance over the phone, or even Zoom, when they might not be set up correctly. [B5]

## Implementation facilitators

### Strong collaborative and interdependent HCP team relationships

A major theme identified as facilitating implementation of the intervention was the importance of strong collaborative and interdependent HCP team relationships. Regular, effective, and clear communication among the IP team members was identified as a key factor for promoting effective IP collaboration. Collaboration between team members at Site A was facilitated by pre-existing working relationships. The intervention effectively leveraged the existing relationships and individual synergies. Site A participants described themselves as “multi-disciplinary” and “trans-disciplinary.” Their trans-disciplinary identity was a source of pride, and they attributed to it their pre-existing familiarity, being “already past that point of awkwardness”. They also acknowledged “really good communication” (A5) and strong working relations. This sense of interdependency and working collaboratively emerged in Site B as well, even though they did not have pre-existing relationships and experienced a higher provider turnover.

There was a sense of security and empowerment in being able to draw on the expertise of colleagues from other professions as needed. In addition to working well within their teams, participants highlighted the importance of collaborating with the HCPs delivering usual care to older adult study participants. Coordination between the intervention team and usual care staff promoted continuity of care particularly during the first 3 months post-discharge, when older adult participants received usual stroke rehabilitation services along with the TCSI.

### Dedicated resources (funding, staffing) to support delivery of the intervention

Another major facilitator to implementing the intervention was that funding was provided to the participating outpatient stroke rehabilitation sites to deliver the intervention. Funding was used to compensate the staff that were delivering the intervention so that they had protected time to deliver the intervention (beyond usual care), as well as offset other material costs associated with the delivery of the intervention. Funding was particularly important to support the Care Coordinator role, which was a new 0.80 FTE position and represented the greatest cost associated with delivery of the intervention. HCPs and Managers highlighted the critical foundational role of the Care Coordinator in leading the IP team, providing the majority of the visits, taking responsibility for the coordination of, and access to, all services, organizing and facilitating the monthly IP team conferences and communication among team members and other stakeholders, establishing goals for each visit, developing and documenting individual care plans, providing regular support to IP team members, acting as a point person for study participants, and managing the collation of online documents.

### Extensive training and ongoing support customized to individual HCP needs

To support intervention fidelity, the HCPs, were provided training by the PI and the RC prior to initiation of the intervention to prepare them for their roles by conveying key intervention activities, research study procedures, and underlying theories. A standardized training manual was developed that includes key content pertaining to all aspects of the intervention. Because the participating HCPs were already experienced in providing usual stroke care, the training focused on expanding their capacity with respect to the new intervention rather than teaching them about usual care at the same time. A key focus in the training was on the management of multimorbidity. Prior to the training, the PI and the RC conducted an informal assessment of the learning needs of the HCPs on each component of the intervention, including the assessment and management of older adults with multimorbidity. Open-ended questions were used to identify training needs, and difficulties and resources relating to the management of multimorbidity in older adults. The results of the needs assessment were used to tailor the training to the individual learning needs of the HCPs regarding delivering the intervention and addressing multimorbidity. Education and role-playing were used to enhance their knowledge and skills in all intervention activities. Training modules were made available online, and further education was provided during the monthly outreach meeting on various topics relevant to the intervention.

As delivery of the TCSI was a learning experience for all involved, ongoing mentoring, education and support of HCPs was an important factor facilitating implementation. Monthly outreach meetings with the HCPs were conducted to enable the PI and the RC to support the intervention teams, monitor intervention implementation, and strategize to address any challenges. Separate, bi-monthly meetings were also held with the Care Coordinators to support them in their roles. Regular research team meetings served as an opportunity to: 1) address clinical-related questions; 2) reinforce and address questions related to implementation of the study (e.g., documentation); 3) provide continuing education for best practices for stroke and stroke rehabilitation, multimorbidity and person-centred care; and 4) share success stories related to positive clinical outcomes and effective collaborative efforts with community partners. Participants felt that the support provided by the research team provided a venue for identifying and addressing issues related to implementation, created a sense of shared ownership among the IP team, and increased HCPs’ comfort and confidence in delivering the intervention. The HCPs valued prompt access to the PI and the RC via phone, email, and in-person to address implementation-related questions.

### Organizational readiness for implementation, strong leadership, and effective local champions

Participants highlighted that alignment of the TCSI with the mission and objectives of their organizations led to high organizational commitment to implementation of the intervention. Participants indicated that the intervention was a priority as it was addressing gaps in transitional care for a segment of the outpatient stroke population with the greatest needs, e.g., older adults with stroke and multimorbidity. Interviews revealed that the daily presence and support of designated Managers and Directors within each of the sites who became effective champions of the intervention was also instrumental in facilitating implementation. They were respected leaders who believed in the project, took ownership, and were committed to keeping the project on track during challenging times. They worked together with the research team and the HCPs to determine how to best operationalize the intervention in their respective clinical settings. This included establishing the IP teams, determining the nature and scope of various roles, designing workflow processes to integrate the intervention into usual practice, identifying information systems for sharing patient-related information within the team, and establishing reporting relationships and responsibilities. These champions attended the monthly research team meetings, provided the IP team members with ongoing support and feedback on the day-to-day implementation of the intervention, and promoted the study within their local setting. They fostered trust and promoted communication among team members in ways that helped to quickly resolve most of the challenges encountered.

### SharePoint and monthly IP team conferences facilitated virtual sharing of information and IP collaboration

HCPs reported that SharePoint served as an effective IP communication tool that facilitated consistent and transparent virtual communication and information sharing among IP team members. SharePoint also served as a platform for sharing training materials, study-related forms, information on community resources, and other study-related materials. Another strategy that facilitated virtual information sharing and IP collaboration was the monthly IP team conferences. The monthly team conferences provided additional opportunities for the team to meet, work cooperatively, benefit from complementary roles, and share responsibility for making decisions regarding patient care. The conferences enabled the HCPs to “get on the same page and work through the goals of the patients and any concerns from the team” [Manager Survey].

### Regular monitoring of intervention implementation, feedback mechanisms and flexibility embedded in implementation led to real-time adaptations increasing adoption

As the intervention progressed over time, barriers to continued adoption occurred that were primarily related to the COVID-19 pandemic. Regular monitoring of intervention implementation, feedback mechanisms and the flexibility embedded in implementation allowed the research team to be responsive to implementation challenges, to identify new implementation strategies, and to ultimately facilitate continued use of the intervention over the course of implementation. We used an audit and feedback strategy, that involved encouraging feedback from HCPs, older adult study participants, and their caregivers, and regular review of documents that provided a record of the intervention components that were delivered. Monthly research team meetings provided a forum for HCPs and their managers to provide feedback and discuss the results of the audit to identify any gaps or challenges to implementation. This data helped to inform ongoing adaptation of the intervention to the local context and ensure consistency and adherence to the intervention protocol.

## Implementation challenges

### COVID-19 related challenges: Recruiting and retaining intervention staff and disruptions to community-based services and supports

The redeployment of intervention team members at Site B to acute inpatient units to manage COVID-19-related needs resulted in turnover of intervention staff, and the need to stop the trial three months early. Turnover of intervention staff resulted in disruptions to the delivery of the intervention, challenges in maintaining steady working relationships among the IP team members, and a lack of continuity of care. Participants reported significant challenges recruiting staff to replace IP team members. Significant time and effort, on the part of the managers and the research team, was devoted to the discussion of recruitment issues, and identifying potential recruitment strategies. This was particularly challenging since a health human resource shortage existed at this site prior to initiating the study. HCPs also spoke of the challenges accessing community-based services, that were negatively impacted by the COVID-19 pandemic. Lockdowns, social distancing measures and restrictions on gatherings affected the delivery of these services and made it challenging to access the services and supports they needed. Many community services pivoted from in-person to virtual delivery or faced financial challenges that led to reduced capacity or cessation of services.

### Poor communication and collaboration with local community-based service partners

HCPs spoke of the challenges of identifying and linking patients to home and community-based services, with primary care physicians being a particularly challenging group to engage. Some patients who were asked to follow up with primary care were unable to do so as they didn’t have a family physician. Many IP team members were not familiar with the community-based programs and services in their region that supported stroke rehabilitation and recovery, and the criteria for acceptance into these programs, and struggled to identify appropriate referrals.

Community resources, including existing programs and services that supported older adults with stroke and multimorbidity, were reviewed during the initial training sessions, posted on SharePoint, and the MSRJ website. The Care Coordinator, and other IP team members were encouraged to learn about the resources in their communities and develop relationships with staff from these organizations. HCPs highlighted the difficulties referring patients to home care services. Even HCPs with decades-long experience in stroke rehabilitation who spoke of their well-established relationships with their community colleagues “we know them by first name if we were to call an organization… to make this referral” (A4) were unclear about the criteria for referring patients to home care”. HCPs confirmed that implementation would be positively affected if the IP team built stronger partnerships with home and community-based service providers and primary care.

### Documentation burden (intervention-related)

HCPs cited the intervention-related screening and assessment tools, and forms, (e.g., visit record, and alerts) as both a facilitator and a challenge; they enhanced assessment and information sharing; yet they were burdensome to implement. HCPs indicated that it was often difficult to conduct assessments and address discipline-specific and patient-related concerns within the time allotted for the visit. Visits took longer than expected, requiring a few to prepare for their visits and complete intervention-related documentation on their own personal time. While SharePoint served as an effective IP communication tool that facilitated virtual communication and information sharing among team members, the data in SharePoint could not be integrated into the EMR. This meant that HCPs frequently needed to document the same information in both SharePoint and the EMR, resulting in increased documentation burden.

HCPs were also concerned that asking patients what may be seen as “really invasive questions” shortly after they had returned home “didn’t get them time to settle and come to the reality that they just had a stroke and that they're coming home now,” while potentially adding to caregiver burden (Site B interview). HCPs also felt that patients thought that screenings were repetitive and lacked clarity and relevance; they were overwhelming and challenging, particularly for those with cognitive impairments.

### Technological/Virtual challenges impeded virtual delivery of the intervention

Virtual delivery of the intervention allowed providers to support patients’ recovery journey during the COVID-19 pandemic that restricted face-to-face visits. Video visits (using Microsoft Teams and Zoom) were seen as most effective. However, one participant observed, “a lot of participants […] don't have the technology or they don't have access to the internet, and some of them don't even have email” (B3). Another participant indicated that “sometimes the patients involved did not have the technology to participate or had technical issues with the connection to our staff” [Manager survey]. Training patients and their caregivers on how to access and use videoconference helped to address some of the technological barriers. An SLP described the difference that a videoconference made to hearing information only over the phone, for instance (B2). More severe strokes, communication impairments, having English as a second language and general technological difficulties made virtual care delivery even more challenging. Some providers also found it difficult to provide virtual support for physiotherapy, exercise management, social and safety concerns, and medication reconciliation. The option to conduct visits by phone or videoconference, coupled with the pre-discharge training offered to patients and their caregivers at Site B on how to use online platforms and related equipment supported the implementation of virtual visits.

## Discussion

This study aimed to enhance understanding of the facilitators and challenges to implementing the TCSI in real-world clinical practice, from the perspective of frontline HCPs and Managers. Our study had several innovative aspects. First, it involved testing a TC intervention that focused on clinically complex older adults (with stroke and multimorbidity) who are often excluded from studies evaluating TC models.^26–29^ Thus, this research enhances our understanding of what is required to successfully implement TC interventions to a population such as this who are most susceptible to health challenges associated with fragmented transitional care. Second, it focused on identifying the facilitators and challenges to implementing the TCSI in real-world settings. Evidence on how to best implement effective, evidence-based transitional care interventions is lacking thereby limiting the successful spread of these interventions to other jurisdictions.^66^ Third, we evaluated the factors influencing implementation of a virtual TC intervention during the COVID-19 pandemic. Findings from studies and systematic reviews suggest that virtual stroke rehabilitation services (also called telemedicine, telehealth, or telerehabilitation) can be both feasible and effective.^67–70^ However, they are based on a weak evidence base, provide little information on implementation, and the preferences of people with stroke, and none focus specifically on older adults with stroke and multimorbidity. Thus, the TCSI study served to address this gap in the literature.

Our study identified an interplay of ten main factors (6 facilitators; 4 challenges) that influenced implementation of this multi-component intervention. Most of these factors (5 facilitators, 2 challenges) were related to five inner setting constructs: relational connections, available resources, access to knowledge and information, leadership engagement and mission alliance, information technology and communications, and compatibility. The finding that inner setting CFIR constructs were most common in influencing implementation means that factors within the organization itself play a significant role in determining the success or failure of the implementation process.^60^ Thus, our results support claims that the context requires attention in itself and not only as a background description of a study.^71^ Five (3 facilitators, 2 challenges) were related to three intervention characteristic constructs: innovation design, innovation adaptability, and design, quality, and packaging. This indicates that specific features and attributes of the intervention itself play an important role in determining the success of its implementation within an organization.^60^ Four (3 facilitators, 1 challenge) were related to four implementation process constructs: teaming, assessing needs and planning, reflecting, and evaluating, engaging intervention deliverers. Factors related to the outer setting and individual characteristics were less reported.

Klaic et al,^72^ in a recent overview of reviews on the implementability of healthcare interventions, found that the implementation and uptake of an intervention by HCPs depends heavily on their perceptions of the intervention. These perceptions are referred to as views about the ‘implementability’ of the intervention, and are considered independent from the objective features of the intervention.^72^ We define the term ‘implementability’ as the likelihood that an intervention will be implemented successfully in a given context.^72,73^ Recent work of Damschroder and colleagues^60^ clarified the relationship between the CFIR determinants, captured in this study, and the implementability of an intervention. They proposed that CFIR determinants inform antecedent assessments (i.e., acceptability, appropriateness, feasibility, implementation climate & readiness), and these in turn inform the implementability of an intervention, which in turn shapes the ultimate success of the intervention (achievement of intervention outcomes). The qualitative evidence captured in our study offered evidence of three antecedent constructs (appropriateness, feasibility, and implementation climate and readiness) described below.

### Appropriateness

Appropriateness is the perceived fit, relevance, or compatibility of the intervention for a given practice setting.^74^ Factors related to the appropriateness of the intervention included alignment of the goals of the intervention and the mission and objectives of the organizations; strong leadership support to enable the change in practice and support HCPs in delivering the intervention; and the ability to integrate the intervention into existing workflows in usual practice.

### Feasibility

This construct captures the extent to which the program is viewed by HCPS and managers as doable, workable, and easy to use/deliver.^73,74^ Factors that enhanced the feasibility of the intervention included strong collaborative and interdependent HCP relationships, dedicated resources (funding, staffing) to support delivery of intervention, formal structures (SharePoint and monthly IP team conferences) that facilitated virtual information sharing, and regular monitoring and feedback mechanisms. Most challenges identified in this study related to the feasibility of implementing the intervention. HCPs and Managers cited COVID-related challenges (recruiting and retaining intervention staff, disruptions to community-based services and supports), intervention-related documentation burden, challenges related to virtual delivery of the intervention, and poor communication and collaboration with local community-based service partners.

### Implementation Climate and Readiness

Readiness refers to the preparedness of an organization or system to successfully implement an intervention.^75^ Factors that enhanced organizational readiness to implement the intervention included organizational readiness to implement the intervention, strong leadership support, effective champions, strong provider team collaboration, dedicated resources to support intervention delivery, regular monitoring, of intervention implementation, feedback mechanisms, and flexibility embedded in implementation, and that all HCPs received extensive training customized to individual HCP needs (including access to online tools) as well as ongoing support.

## Key implications

Overall, these findings on the appropriateness, feasibility and implementation climate and readiness suggest that implementing the TCSI is connected to the following health policy priorities:

### Population segmentation

In Ontario (Canada) and globally segmenting the population into subgroups with shared needs is consistent with current health policy directions. Population segmentation is a promising approach for enabling the development and evaluation of integrated models of care that meet the healthcare needs of selected target populations with the greatest potential for significant impact.^76^ The World Health Organization identified reaching underserved and marginalized populations as an important approach for the delivery of integrated, equity-oriented person-centred health services. The participating organizations and policy makers in this study were committed to identifying strategies to improve access to quality health services for underserved and vulnerable populations, such as older adults with stroke and multimorbidity.^77^ Initial and ongoing collaboration and engagement with healthcare decision-makers helped to ensure that the intervention was aligned with the policy context to enhance buy-in and the potential for scaling-up the intervention.^60,75,78–82^

### Management of multimorbidity

#### Strong collaborative and interdependent HCP team relationships

The evidence is clear that the management of older adults with multimorbidity is best addressed by an IP team with strong IP collaboration.^83,84^ Our study finding that strong collaborative and interdependent HCP team relationships facilitated implementation, is consistent with previous studies on TC^80,85^and multimorbidity interventions.^48,86^ Supporting the findings of previous studies, strong collaboration is characterized as cooperative and interdependent working relationships across team members,^82,87,88^ the ability to communicate openly, clearly and effectively, strong coordination of care,^89^ the capacity to share decision-making and solve problems together,^90^ and a partnership guided by patients’ goals and priorities for a high-functioning team.^85^ The presence of strong IP collaboration facilitates the delivery of integrated, person-centred services that are more likely to meet the complex needs of older adults with stroke and multimorbidity.^83,91,92^ Findings suggest that provider team collaboration can be enhanced and reinforced by selecting a provider group with pre-existing relationships, co-locating the team,^86^ maintaining a consistent provider team to support relationship-building, and including training focused on improving IP communication and coordination between team members. The aim of IP education would be to promote collaborative practice, with the goal of improving patient outcomes.^4,93,94^

#### Mechanisms to facilitate IP collaboration and communication

In general**,** considerable evidence suggests that the establishment of systems and processes to effectively manage information and communication positively influences implementation success.^85^ Studies specific to TC and multimorbidity interventions report that the use of a supportive electronic system, such as SharePoint, facilitated implementation by enabling virtual communication and shared information documentation.^5,80,83,86^ Our study is consistent with these findings and suggests that such a system should be designed to enable health care professionals to securely communicate with each other, share patient information, updates, and requests for consultation at all points of access in the system, to ensure seamless transitions in care across the continuum.^5^^,^^95^

#### Dedicated Care Coordinator within the IP team

Previous studies of TC and multimorbidity interventions highlight the importance of a dedicated Care Coordinator to support IP collaboration, coordinate care for individuals, health services and providers, and coordinate care within and beyond the health sector.^48,77,86,96^ Our study is consistent with these findings, and further suggests that the Care Coordinator role is not focused on a single activity, but rather a range of strategies designed to achieve better continuity of care, and enhance the patients’ experience with services, particularly during care transitions.^77,96^ A testament to the perceived importance of this role was that one of the sites created a new Care Coordinator position following completion of the study.

#### Training customized to individual HCP needs and providing ongoing support

Previous studies of TC and multimorbidity interventions have highlighted the importance of extensive provider training (including access to online tools) to prepare them for their roles prior to initiation of the intervention.^78,80,^^97^ Our study and others further highlight the importance of customizing the training to the needs and characteristics of the HCPs and setting.^86^ One way to identify these needs is by assessing skills gaps, which involves evaluating the current knowledge and competencies of HCPs with respect to the different components of the intervention to determine areas where additional training is required. HCPs in our study reported a strong need for education and training specific to person-centred care, patient complexity and the management of stroke in the context of multimorbidity. This finding may have been due to the fact that current best practice guidelines for stroke rehabilitation provide limited recommendations for those with multimorbidity.^33^ Therefore, the initial standardized training for the TCSI should include: 1) strengths based practice to support self-management, 2) a shift from providing disease-specific care to person-centred care and the development of care plans that account for all chronic conditions , and 3) comprehensive assessment (including social determinants of health and treatment burden) and the use of validated tools.^48,98,99^

Findings also confirm evidence from the literature that ongoing and timely support and coaching from managers, senior leadership, and the research team was seen as highly valuable to support the intervention teams, provide continuing education on best practices for the management of stroke and multimorbidity, monitor intervention implementation, and resolve implementation challenges.^86,100^ Consistent with the findings of previous studies, the crucial role of strong communication and feedback between HCP and Managers and the research team highlights the benefits of having organized communication and feedback structures.^101^ Monthly research team meetings provided a structure that supported two-way communication and enabled the research team to engage with the HCPs and Managers by soliciting their input and responding to their needs and concerns. In addition to receiving ongoing support and feedback, the findings suggest that HCPs need to be organized around teams and supported with clear roles and expectations, guidelines, and protected time to deliver the intervention.

### Strengthening community-based partnerships

Consistent with findings of previous TC and multimorbidity interventions, HCPs and Managers in our study spoke of the challenges of engaging with primary care providers in health care system innovations^102,103^ and developing and sustaining community/cross-sector provider relationships.^96^ It is important to recognize that, while COVID was a significant barrier to establishing and maintaining these partnerships during our study, the need to strengthen these relationships is a general requirement that exists outside the pandemic context. These communications and partnerships are fundamental for ensuring the coordinated delivery of health and social services for older adults with stroke and multimorbidity.^5,15,86,96,104^ Our results also highlight the importance of including more information on community-based services in the training program, and the need for early and concerted upfront investments in relationship building between outpatient stroke rehabilitation HCPs and community service partners. This is particularly important for the Care Coordinator who has a prominent role providing system navigation by connecting patients and their caregivers to appropriate services and supports in the broader health care system and the community.

### Strengthening information systems and knowledge management

Study findings corroborate evidence for the importance of continuous monitoring and evaluation of the intervention using specific and measurable objectives to inform ongoing adaptation of the intervention to the local context and support intervention fidelity.^5,77,80,105^ In our study, regular review of the implementation process and outcomes enabled the HCPs and Managers to proactively identify potential issues and make necessary adjustments to ensure the success and effectiveness of the intervention, particularly during the pandemic when the practice setting was constantly changing.

A key source of data for monitoring implementation of the intervention was intervention-specific documentation completed by the HCPs. However, HCPs cited documentation burden (intervention-related) as a major challenge to implementation of the intervention. HCPs reported high levels of dissatisfaction with documentation, significant issues with duplication and redundancy, and the need to use their own personal time to complete documentation. Findings suggest that documentation burden for HCPs could be reduced by integrating the IP communication system (e.g., SharePoint) with other healthcare systems (e.g., EMR). Oorganizations’ seeking to implement the intervention should work collaboratively with HCPs to conduct a comprehensive evaluation of all documentation currently in use in usual care and the intervention to identify areas of duplication and redundancy and determine aspects of documentation that could be modified. Findings also highlight the need to select and embed key metrics in the EMR to track implementation and real-time data collection systems; (so it is not an add-on but a rather, data on a key set of measures routinely collected in everyday practice). There is increasing evidence for the development of information systems, an organizational culture that supports ongoing monitoring and evaluation, knowledge sharing, and using data to track implementation to inform ongoing decision-making and ensure successful implementation of complex, person-centred interventions.^77^

### Support for virtual care delivery

HCPs cited virtual delivery of the intervention as both a facilitator and a challenge; it enabled access to services during the COVID-19 pandemic, yet reduced or challenged access for others, (e.g., those without computers, internet access, technical expertise). Other studies have documented similar challenges with virtual delivery of stroke rehabilitation.^12,106,107^ These challenges are compounded by the lack of evidence on how to effectively implement virtual care in outpatient stroke rehabilitation practice.^108^ The findings from this study may serve as an example of a feasible and effective approach to the delivery of virtual stroke rehabilitation to address this research gap. Our findings add to a rapidly expanding volume of literature that suggests that virtual stroke rehabilitation interventions are feasible and have positive effects on stroke survivors’ rehabilitation^109–111^ and health and self-management outcomes^109,112^ compared with in-person care. Moreover, there is increasing evidence for the effectiveness of virtual approaches to chronic disease management.^106,113^

Our results highlight the need to provide HCPs with specific training and ongoing support on how to clinically navigate a virtual visit, as well as access to on-site support for managing technological issues. However, future research is needed to determine how to best train HCPs in the delivery of virtual care. Although the TCSI in this study was only offered virtually due to pandemic-related restrictions, virtual care may not be appropriate for some patients. Health care organizations planning to deliver the TCSI should consider incorporating tools, such as the Canadian Stroke Network’s virtual care decision framework,^106^ to support HCPs decision-making regarding the most appropriate mode of care delivery, (e.g., virtual, in-person, hybrid).

### Strengths and limitations

A key strength of the study was its pragmatic design. The TCSI trial was designed to be highly pragmatic using the criteria described in the Pragmatic Explanatory Continuum Indicator Summary Version 2 (PRECIS-2) tool.^114,115^ A primary goal of pragmatic trials is to rapidly influence clinical decision-making and policy. As such, the TCSI trial allowed flexibility in the implementation of the intervention as it occurs in real-world settings, and among participants representative of the population presenting in the outpatient clinic, and places little control on the heterogeneity present within usual care.^114,115^ Thus, the TCSI study provides a realistic indicator of TCSI implementation for older adults with stroke and multimorbidity. Another strength of the study was the use of the CFIR framework to guide the development of data collection tools (e.g., interview and focus group guides), data extraction (e.g., team meeting minutes), and the analysis. Using CFIR to guide the study enabled us to build on the existing knowledge about implementation science to explore determinants or factors at multiple levels of influence on the implementation of a transitional care intervention for older adults with multimorbidity and stroke. Data collection included 12 HCPs and 3 Managers, who provided rich experiences, and data saturation was reached in both settings. Other strengths of this study included the use of a rigorous qualitative design, and the use of multiple qualitative data generation approaches (structured interviews, focus groups) to enable deep exploration of the subject matter both individually and collectively.

This study has several limitations that point to potential directions for future research. First, the study was conducted in two regions in Ontario and thus may not be transferable to other settings. Second, the study had a relatively small sample size, and the facilitators and challenges described in the present study are based on the participants’ descriptions of their practice, competence, and experience. Their experiences might not be representative of outpatient stroke rehabilitation services in other settings. A future RCT should involve multiple sites, to explore implementation facilitators and challenges across a broader range of settings and contexts. Third, the study was conducted during the COVID-19 pandemic. Despite our efforts to minimize the effects of the pandemic, we do not know the impact that the pandemic had on the results of the study or what may be different if the study was conducted under ‘normal’ circumstances, e.g., not under the constraints of the pandemic.

Fourth, our study was not designed to establish causal inferences, but rather to provide a broad as well as in-depth understanding of the factors that influence implementation of the TCSI across different outpatient stroke rehabilitation settings. Future work can build on the groundwork laid by our initial investigation to draw causal conclusions about factors that facilitate or inhibit effective delivery of the TCSI. A final limitation is that while attempts were made to gather information from older adult and caregiver participants regarding the implementation of the TCSI, because of the participants’ complex health conditions, very few agreed to participate in this component of the study. As a result, this study is missing this important perspective.

## Conclusion

This research enhances understanding of the myriad of factors influencing implementation of the TCSI, and the conditions under which implementation is more likely to succeed (e.g., strong collaborative and interdependent HCP relationships; dedicated resources to support delivery of the intervention; organizational readiness for implementation, strong leadership, and effective local champions). Addressing these factors with active and continual engagement of patients, HCPs, and health system partners presents the most promising approach to optimize the implementability of the TCSI into real-world practice to improve health outcomes and enhance the quality and experience of care transitions for clinically complex older adults with stroke and multimorbidity.

## Supplemental Material

Supplemental Material - Implementation of the virtual transitional care stroke intervention for older adults with stroke and multimorbidity: A qualitative descriptive study Supplemental Material for Implementation of the virtual transitional care stroke intervention for older adults with stroke and multimorbidity: A qualitative descriptive study by Maureen Markle-Reid, Kathryn Fisher, Kimberly M. Walker, Jill I. Cameron, David Dayler, Rebecca Fleck, Amiram Gafni, Rebecca Ganann, Ken Hajas, Barbara Koetsier, Robert Mahony, Chris Pollard, Jim Prescott, Tammy Rooke, Carly Whitmore in Journal of Multimorbidity and Comorbidity

## Appendix

### Abbreviations

TCSI: Transitional Care Stroke Intervention
IP: Interprofessional
TC: Transitional Care
CFIR: Consolidated Framework for Implementation Research
COVID-19: Coronavirus Disease
REB: Research Ethics Board
RCT: Randomized Controlled Trial
PT: Physical Therapist
OT: Occupational Therapist
SLP: Speech Language Pathologist
RPN: Registered Practical Nurse
RN: Registered Nurse
RA: Research Assistant
RC: Research Coordinator
HCP: Health Care Provider
PI: Principal investigator
EMR: Electronic medical record
