# Supplementary material for: Implementation of the virtual transitional care stroke intervention for older adults with stroke and multimorbidity: A qualitative descriptive study
Source: J Multimorb Comorb. 2025 Feb 26;15:26335565251323748. doi: 10.1177/26335565251323748 (PMC11863252; doi:10.1177/26335565251323748)
Supplement: Supplemental Material - Implementation of the virtual transitional care stroke intervention for older adults with stroke and multimorbidity: A qualitative descriptive study [file sj-pdf-1-cob-10.1177_26335565251323748.pdf]

Implementation of the Virtual Transitional Care Stroke Intervention for Older Adults with Stroke and Multimorbidity: A Qualitative Descriptive Study

Supplement Appendix 1. List of Chronic Conditions

**Cardiovascular**
Atrial fibrillation
Hyperlipidemia (cholesterol problem)
Coronary artery disease (ischemic heart disease, angina, previous heart attack)
Congenital malformed valve, valve heart disease or replacement
Hypertension (high blood pressure)
Heart failure (including heart valve disease or replacement)
Peripheral vascular disease
Stroke (cerebrovascular accident or transient ischemic attack)
**Respiratory**
Asthma
COPD (chronic obstructive pulmonary disorder), chronic bronchitis, emphysema
Pulmonary fibrosis (or bronchiectasis)
Other lung conditions (e.g. pulmonary fibrosis cystic fibrosis)
**Mental/Mood Disorders**
Depression
Anxiety
Schizophrenia or bipolar disease
Anorexia or bulimia
**Gastrointestinal**
Stomach problem (e.g. gastric reflux or peptic ulcer symptoms)
Colon problem (e.g. chronic inflammatory disease, irritable bowel syndrome, or diverticulitis)
Dyspepsia (chronic indigestion)
Constipation
Bowel obstruction
**Endocrine**
Diabetes
Thyroid disorders
**Liver**
Chronic liver disease (including chronic hepatitis or cirrhosis)
**Kidney and Urogenital Disorders**
Chronic kidney disease or failure (including other kidney disease i.e. kidney stones, alport syndrome, kidney leaking, etc.)
Recurrent urinary tract infection
Bladder problems (including cystitis, prolapse or repair)
Bladder incontinence (does not need to be confirmed by a doctor)
Fecal incontinence (does not need to be confirmed by a doctor)
Gout
Prostate disorders
Sexual disorder (including erectile dysfunction)

**Hearing and Vision**
Blindness and low vision
Glaucoma
Macular degeneration, diabetic retinopathy, Fuchs disease, and other vision disorders
Hearing loss (hearing problems and vestibular disorders)
**Neurological**
Alzheimer disease or another form of dementia
Peripheral neuropathy (nerve damage)
Epilepsy
Multiple sclerosis
Parkinson’s disease
**Musculoskeletal**
Arthritis/ osteoarthritis/ osteoporosis
Rheumatoid arthritis, other inflammatory and systemic connective tissue disorders
**Pain**
Painful condition (i.e. chronic back pain, fibromyalgia, tendonitis, bursitis, etc.)
Migraine
**Substance abuse**
Alcohol problems
Other substance misuse
**Infection**
Chronic sinusitis
HIV
**Other**
Cancer in the past 5 years (including melanoma, but not other skin cancers; including precancerous cells)
Blood disorders (including anemia and low red blood cell count)
Psoriasis or eczema
Sleep wake disorders (including insomnia, sleep apnea, narcolepsy)

Consolidated criteria for reporting qualitative studies (COREQ): 32-item checklist

**No. Item**	**Guide questions/description**	**Reported on Page #**
**Domain 1: Research team and reflexivity**		
Personal Characteristics		
1. Interviewer/facilitator	Which author/s conducted the interview or focus group?	p.11
2. Credentials	What were the researcher’s credentials? E.g. PhD, MD	p. 11
3. Occupation	What was their occupation at the time of the study?	p. 11
4. Gender	Was the researcher male or female?	p. 11
5. Experience and training	What experience or training did the researcher have?	p. 11
Relationship with participants		
6. Relationship established	Was a relationship established prior to study commencement?	p. 11
7. Participant knowledge of the interviewer	What did the participants know about the researcher? e.g. personal goals, reasons for doing the research	p. 11
8. Interviewer characteristics	What characteristics were reported about the interviewer/facilitator? e.g. Bias, assumptions, reasons and interests in the research topic	p. 11

**Domain 2: study design**		
Theoretical framework		
9. Methodological orientation and Theory	What methodological orientation was stated to underpin the study? e.g. grounded theory, discourse analysis, ethnography, phenomenology, content analysis	p. 9-10
Participant selection		
10. Sampling	How were participants selected? e.g. purposive, convenience, consecutive, snowball	p. 10
11. Method of approach	How were participants approached? e.g. face-to-face, telephone, mail, email	p. 11
12. Sample size	How many participants were in the study?	p. 13
13. Non-participation	How many people refused to participate or dropped out? Reasons?	N/A
Setting		
14. Setting of data collection	Where was the data collected? e.g. home, clinic, workplace	p. 11
15. Presence of non-participants	Was anyone else present besides the participants and researchers?	N/A
16. Description of sample	What are the important characteristics of the sample? e.g. demographic data, date	p. 13-14, Table 1
Data collection		
17. Interview guide	Were questions, prompts, guides provided by the authors? Was it pilot tested?	p. 11
18. Repeat interviews	Were repeat interviews carried out? If yes, how many?	p. 11
19. Audio/visual recording	Did the research use audio or visual recording to collect the data?	p. 12
20. Field notes	Were field notes made during and/or after the interview or focus group?	p. 13
21. Duration	What was the duration of the interviews or focus group?	p. 11
22. Data saturation	Was data saturation discussed?	p. 32
23. Transcripts returned	Were transcripts returned to participants for comment and/or correction?	N/A
**Domain 3: analysis and findings**		
Data analysis		

24. Number of data coders	How many data coders coded the data?	p. 12
25. Description of the coding tree	Did authors provide a description of the coding tree?	
26. Derivation of themes	Were themes identified in advance or derived from the data?	p. 12-13
27. Software	What software, if applicable, was used to manage the data?	p. 12
28. Participant checking	Did participants provide feedback on the findings?	N/A
Reporting		
29. Quotations presented	Were participant quotations presented to illustrate the themes/findings? Was each quotation identified? e.g. participant number	p. 15-22, Table 2
30. Data and findings consistent	Was there consistency between the data presented and the findings?	p. 15-22
31. Clarity of major themes	Were major themes clearly presented in the findings?	p. 15-22
32. Clarity of minor themes	Is there a description of diverse cases or discussion of minor themes?	N/A
